# Mechanistic study of potentially toxic element removal from water by waste putty powder-derived hydroxyapatite

**DOI:** 10.1039/d6ra07232a

**Published:** 2026-08-20

**Authors:** Yonghong Zheng, Ziyang Zhang, Zhiguo Zhang, Weihua Peng, Yi Liu, Xueting Zhen, Mingxia Huang

**Affiliations:** a Anhui Province Engineering Laboratory of Water and Soil Resources Comprehensive Utilization and Ecological Protection in High Groundwater Mining Area, Anhui University of Science and Technology Huainan 232001 China; b School of Earth and Environment, Anhui University of Science and Technology Huainan 232001 China zzgaust@126.com +86-554-6651501; c Key Laboratory of Mine Water Resource Utilization of Anhui Higher Education Institutes, Suzhou University Suzhou 234000 China

## Abstract

Waste putty powder-derived hydroxyapatite (P-HAP) was synthesized *via* a chemical precipitation method using waste putty powder as the calcium source. The adsorption performance and mechanisms of P-HAP for the removal of potentially toxic elements, including Cd(ii), Pb(ii), and Cr(vi), from aqueous solutions were investigated. The underlying adsorption mechanisms were elucidated using XRD, SEM, EDS, and FT-IR analyses. The results showed that: ① under a P-HAP dosage of 1.6 g L^−1^ and a temperature of 25 °C, the adsorption capacity of P-HAP for Pb(ii) reached 62.50 mg g^−1^, with a removal efficiency of 100%, at an initial Pb(ii) concentration of 100 mg L^−1^ and pH 4. Under an initial Cd(ii) concentration of 100 mg L^−1^ and pH 10, the adsorption capacity for Cd(ii) was 40.63 mg g^−1^, with a removal efficiency of 65.00%. For Cr(vi), at an initial concentration of 10 mg L^−1^ and pH 4, the adsorption capacity was 1.43 mg g^−1^, corresponding to a removal efficiency of 22.94%. ② The adsorption behavior of the three potentially toxic elements (PTEs) was better described by the Langmuir isotherm and pseudo-second-order kinetic models. The adsorption of Pb(ii) and Cd(ii) onto P-HAP primarily occurred through dissolution–precipitation, surface complexation, and ion exchange, whereas the adsorption of Cr(vi) was dominated by surface complexation. In addition, electrostatic attraction has a synergistic effect on the adsorption process.

## Abstract 2

Waste putty powder-derived Hydroxyapatite (P-HAP) was synthesized *via* a chemical precipitation method using waste putty powder as the calcium source.

## Graphical abstract

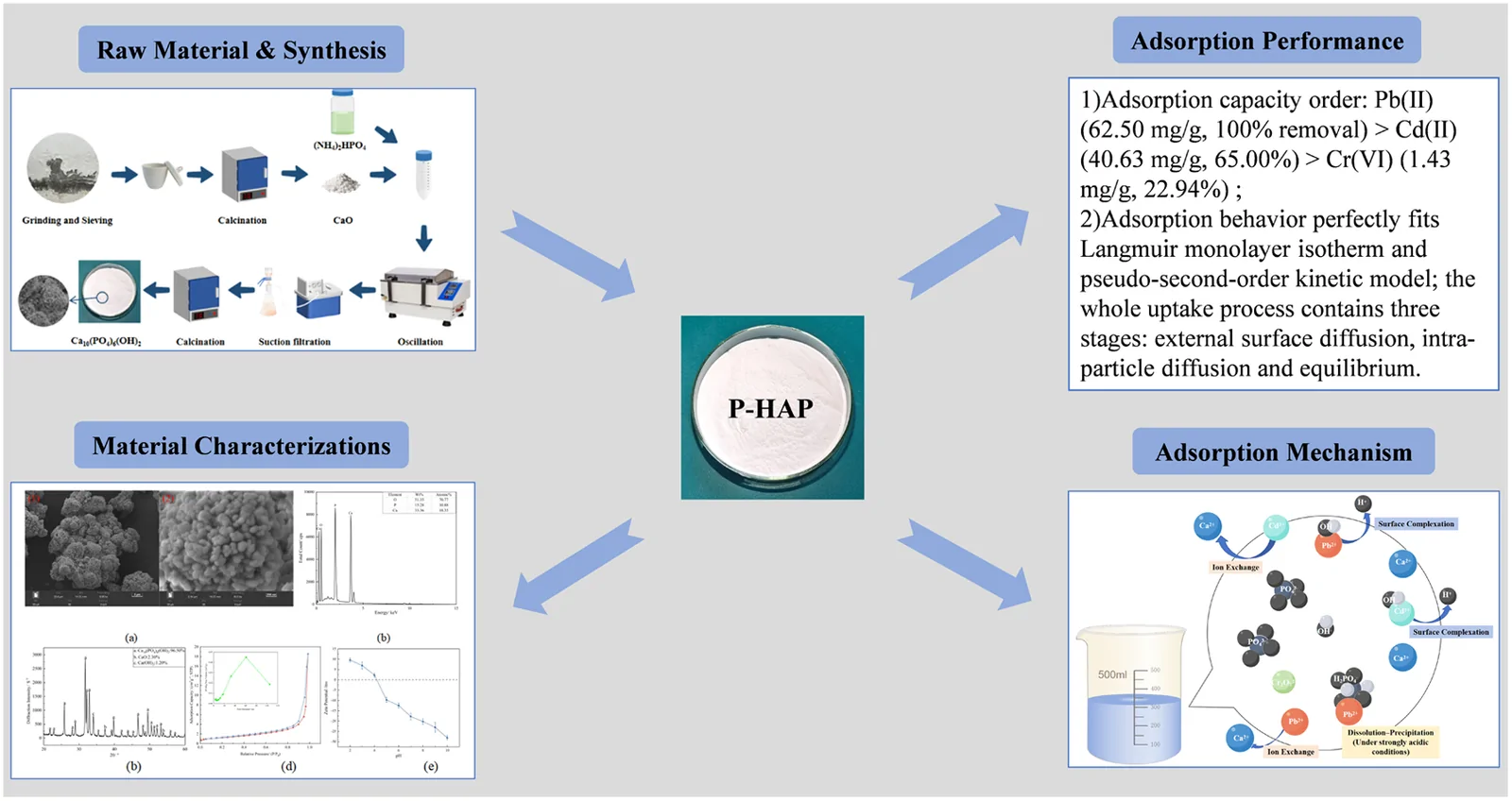

## 1 Introduction

Potentially toxic elements (PTEs) in aquatic environments have attracted widespread attention because of their high toxicity, non-biodegradability, and tendency to bioaccumulate. Among them, Pb(ii) can damage the nervous system and impair renal function, Cd(ii) can cause kidney injury and skeletal disorders, and Cr(vi) exhibits strong toxicity and carcinogenicity.^1,2^ Therefore, developing efficient technologies for the removal of PTEs is of great significance for protecting both the ecological environment and human health. Currently, commonly used water treatment technologies include chemical precipitation, ion exchange, membrane filtration, electrochemical methods, and adsorption. Chemical precipitation is simple and cost-effective but generates large amounts of sludge.^3^ Ion exchange provides high selectivity but requires frequent regeneration.^4^ Membrane filtration achieves high removal efficiency but is limited by membrane fouling and high operating costs.^5^ Electrochemical techniques are highly effective but energy-intensive.^4^ In contrast, adsorption offers several advantages, including simple operation, low cost, high removal efficiency, and broad applicability.^6^ Consequently, it has become one of the most widely adopted water treatment technologies.^7–10^

The removal efficiency of pollutants from aqueous systems through adsorption largely depends on the properties of the adsorbent.^11^ Hydroxyapatite (HAP) has attracted considerable attention for the remediation of heavy metal-contaminated water because of its unique crystal structure, low solubility, abundant surface functional groups, and excellent ion-exchange capacity.^12^ Previous studies have shown that the adsorption of PTEs by HAP primarily involves ion exchange, dissolution–precipitation, surface complexation, and electrostatic interactions.^13^ Among these mechanisms, ion exchange between Ca^2+^ and PTEs, together with the formation of insoluble metal phosphate precipitates, is considered the primary pathway for PTEs removal. Therefore, developing low-cost, high-performance HAP materials and elucidating their adsorption performance and underlying mechanisms are of great significance for the control of PTEs pollution.

To date, numerous studies have reported the excellent adsorption performance of HAP for the removal of various PTEs. For example, Mobasherpour *et al.* reported that nano-hydroxyapatite exhibits high adsorption capacities for Pb(ii), Cd(ii), and Ni^2+^.^14^ Silviu *et al.* prepared a hydroxyapatite/sodium bicarbonate (HAP–SB) composite with high removal efficiency for Pb(ii).^15^ Shafqaat *et al.* demonstrated that metal-doped hydroxyapatite possesses excellent adsorption capacity for a wide range of metal ions.^16^ However, most previous studies have focused on synthesizing HAP from high-purity chemical reagents or biomass wastes (such as eggshells, animal bones, and shells). In contrast, the use of construction waste as an alternative calcium source for HAP synthesis and its application in the remediation of heavy metal-contaminated water has received relatively little attention.

In recent years, the rapid advancement of urbanization in China has led to a continuous increase in construction waste generation, with the cumulative stock exceeding 2 × 10^10^ t and the annual increase reaching approximately 3.5 × 10^9^ t, accounting for about 40% of total municipal solid waste.^17^ Current research on the recycling and utilization of construction waste has primarily focused on recycled coarse and fine aggregates, whereas the resource utilization of powder-based waste components remains relatively limited.^18^ Waste putty powder, a typical fine powder component of construction waste, is primarily composed of calcium carbonate and is characterized by wide availability, low cost, and abundant calcium content. Compared with biomass-derived calcium sources, such as eggshells and bone powder, waste putty powder is generated in larger quantities, is easier to collect, and offers greater potential for the simultaneous reduction and resource utilization of construction waste. Therefore, waste putty powder represents a promising but underutilized calcium source derived from construction waste.

In this study, P-HAP was synthesized *via* a chemical precipitation method using waste putty powder derived from construction debris as the calcium source.^13^ The adsorption performance of P-HAP toward Cd(ii), Pb(ii), and Cr(vi), as well as the removal mechanisms, was systematically investigated. This study provides theoretical insights and technical guidance for the resource utilization of calcium-rich construction waste and the advanced treatment of heavy metal-contaminated water.

## 2 Materials and methods

### 2.1 Materials and instruments

#### 2.1.1 Reagents

Diammonium hydrogen phosphate ((NH_4_)_2_HPO_4_, AR, Xilong Scientific Co., Ltd, China), nitric acid (HNO_3_, GR, Sinopharm Chemical Reagent Co., Ltd, China), ammonium hydroxide (NH_4_OH, GR, Sinopharm Chemical Reagent Co., Ltd, China), cadmium nitrate tetrahydrate (Cd(NO_3_)_2_·4H_2_O, AR, Tianjin Damao Chemical Reagent Factory, China), lead chloride (PbCl_2_, AR, Tianjin Fuchen Chemical Reagent Factory, China), and potassium dichromate (K_2_Cr_2_O_7_, GR, Shanghai Lingfeng Chemical Reagent Co., Ltd, China).

#### 2.1.2 Instruments and equipment for material preparation

High-speed pulverizer (FW400A, Beijing Kewei Yongxing Instrument Co., Ltd, China), microwave muffle furnace (CY-MU1400C-S, Hunan Changyi Microwave Technology Co., Ltd, China), constant-temperature water bath shaker (SHZ-82A, Jintan Tianjing Experimental Instrument Factory, China), and pH meter (FE28, Mettler-Toledo Instruments (Shanghai) Co., Ltd, Switzerland).

#### 2.1.3 Instruments and equipment for material characterization and solute concentration determination

X-ray diffractometer (XRD, Rigaku SmartLab, Rigaku Corporation, Japan), scanning electron microscope (SEM, TESCAN MIRA4, TESCAN Group, Czech Republic), energy dispersive X-ray spectrometer (EDS, OXFORD Xplore 30, Oxford Instruments, Germany), Fourier transform infrared spectrometer (FTIR, Nicolet IS50, Thermo Fisher Scientific, USA), inductively coupled plasma optical emission spectrometer (ICP-OES, Avio 550 Max, PerkinElmer Management (Shanghai) Co., Ltd, China), automatic specific surface area and porosity analyzer (AUTOSORB IQ, Quantachrome Instruments, USA), nanoparticle laser particle size and zeta potential analyzer (90Plus PALS, Brookhaven Instruments Corporation, USA), and X-ray fluorescence spectrometer (XRF, OLYMPUS Vanta, USA).

### 2.2 Preparation of P-HAP

Waste putty powder was collected from a construction site in Huainan, and its chemical composition is presented in Table 1. The collected waste putty powder was first crushed using a high-speed pulverizer and then sieved through a 0.075 mm (200 mesh) sieve. The sieved powder was placed in a ceramic crucible and calcined in a microwave muffle furnace at 450 °C for 2 h, followed by further calcination at 900 °C for 4 h. During the calcination process, calcium carbonate (CaCO_3_) in the waste putty powder was decomposed into calcium oxide (CaO), producing a white powder that was subsequently ground to obtain a fine CaO powder.

**Table 1:** Chemical compositions of the products obtained at each stage of the preparation process

	Chemical compositions	Content (%)
Waste putty powder	CaCO_3_	94.20
	MgCO_3_	2.30
	SiO_2_	0.52
	Al_2_O_3_	0.48
	Other	2.50
The product after the first calcination	CaO	90.01
	MgO	1.93
	SiO_2_	0.91
	Al_2_O_3_	0.84
	Other	6.31

After screening the key variables through single-factor experiments, Box–Behnken response surface optimization was carried out using Design Expert software. A four-factor, three-level experimental design was adopted, with solution pH (9, 9.5, 10), oscillation temperature (65, 70, 75 °C), oscillation time (2.5, 3, 3.5 h), and CaO particle size (400, 600, 800 mesh) as the factors, to determine the optimal preparation process parameters: solution pH of 9.43, reaction temperature of 71 °C, reaction time of 2 h 53 min, CaO particle size of 600 mesh, calcination temperature of 800 °C, and calcination time of 3 h. Three parallel experiments were performed under the optimized conditions.

**Fig. 1:**
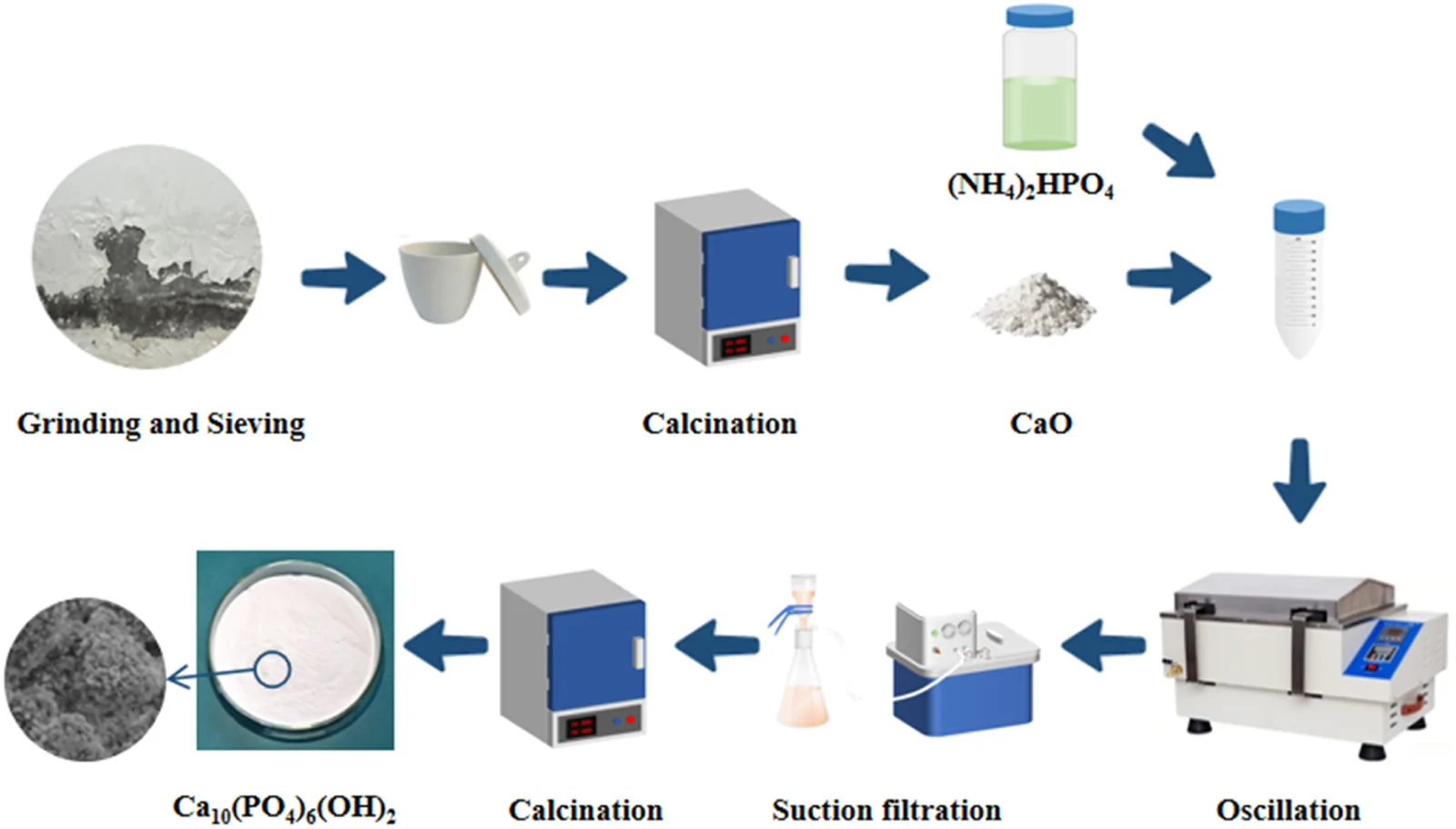
Flowchart for P-HAP preparation.

A 0.5 mol per L diammonium hydrogen phosphate solution was prepared using ultrapure water, and its pH was adjusted to 9.43 using aqueous ammonia and dilute nitric acid. CaO powder was then added to the solution at a Ca/P molar ratio of 1.67. The resulting suspension was transferred to a thermostatic water-bath shaker and reacted at 71 °C with a shaking speed of 180 rpm for 2 h 53 min. After the reaction, the solid product was washed three times with ultrapure water and allowed to settle, after which the supernatant was removed by filtration through a 0.45 µm membrane. The recovered solid was subsequently calcined in a microwave muffle furnace at 800 °C for 3 h to obtain P-HAP. The chemical compositions of the products obtained at each stage of the preparation process are presented in Table 1, and the schematic flowchart of the P-HAP synthesis is shown in Fig. 1.

### 2.3 Adsorption performance experiments

A specified amount of the prepared P-HAP and 25 mL of contaminant-containing solution were added to a 50 mL centrifuge tube. The suspension then oscillated in a digital thermostatic water-bath shaker at 180 rpm and 25 °C. After oscillation for a predetermined period, the suspension was filtered through a 0.45 µm membrane filter, and the concentrations of Cd(ii), Pb(ii), and Cr(vi) in the filtrate were determined using an inductively coupled plasma optical emission spectrometer according to the standard method HJ 776-2015. The initial concentration gradients of the three PTEs solutions are presented in Table 2.

**Table 2:** Initial concentrations of the three PTEs solutions

Types of PTEs	Initial concentration gradient (mg L^−1^) of PTEs solutions							
Cd(ii)	5	10	15	20	40	60	80	100
Pb(ii)	5	10	15	20	40	60	80	100
Cr(vi)	0.5	1	1.5	2	4	6	8	10

#### 2.3.1 Effect of P-HAP dosage and solution pH

The P-HAP dosage was varied at 0.40, 0.80, 1.20, 1.60, 2.40, 3.20, and 4.00 g L^−1^, with the initial pollutant concentrations set at the maximum values listed in Table 2. All other experimental conditions were kept constant (pH = 7, 180 rpm, 25 °C) to investigate the effect of P-HAP dosage on pollutant adsorption performance.

The pH values of the solutions were adjusted to 4, 5, 6, 7, 8, 9, and 10, while the initial pollutant concentrations were set at the maximum values listed in Table 2. All other experimental conditions were maintained constant (180 rpm, 25 °C, 1.60 g per L P-HAP dosage) to investigate the effect of solution pH on pollutant adsorption performance.

The adsorption capacity (*Q*_e_, mg g^−1^) (1) and adsorption rate (*R*, %) (2) were calculated using the following equations:1
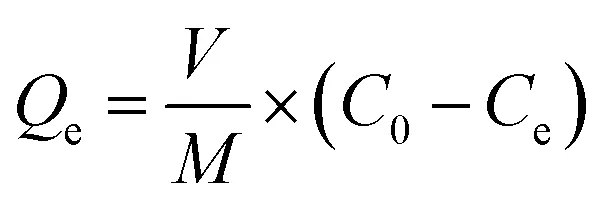
2
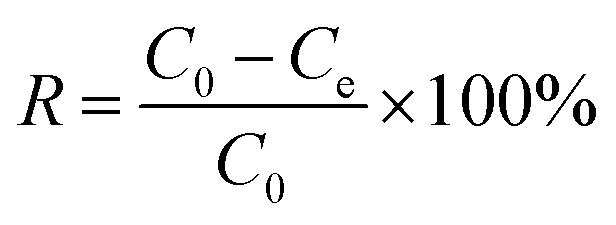
where *C*_0_ (mg L^−1^) represents the initial concentration of contaminants in the solution, *C*_e_ (mg L^−1^) shows the equilibrium concentration of contaminants in the solution, *V* (L) signifies the solution volume, and *M* (g) represents the mass of P-HAP.

#### 2.3.2 Adsorption isotherm experiments

The initial concentration gradients of the three PTE solutions were designed as shown in Table 2. All other experimental conditions were maintained constant (pH = 7, 180 rpm, 25 °C, 1.60 g per L P-HAP). The adsorption isotherm experiments were conducted to investigate the interaction between P-HAP and the target pollutants and to further elucidate the adsorption behavior of each pollutant on the surface of P-HAP.

The experimental adsorption data were fitted using three adsorption isotherm models: Langmuir (3), Freundlich (5), and Temkin (6). The favorability of the adsorption process was evaluated by calculating the Langmuir separation factor (*R*_L_) (4). The equations for these three models are as follows:3
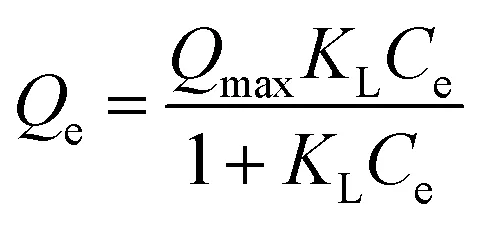
4
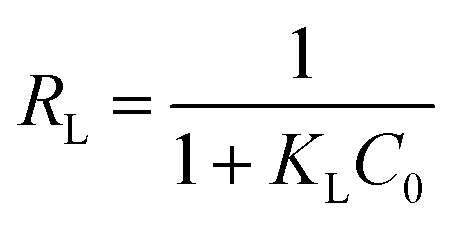
5
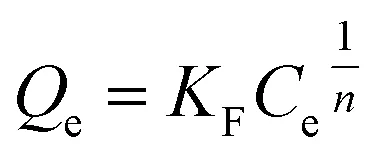
6
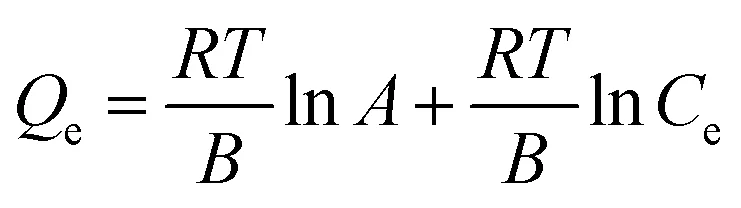
where *C*_e_ (mg L^−1^) and *Q*_e_ (mg g^−1^) represent the equilibrium concentration of the pollutant in solution and the equilibrium adsorption capacity of P-HAP, respectively; *Q*_max_ (mg g^−1^) represents the theoretical maximum adsorption capacity of P-HAP for the pollutant; *R*_L_ is a dimensionless Langmuir separation factor; *K*_L_ is the Langmuir adsorption constant; *K*_F_ and 1/*n* are the Freundlich constants; *R* is the universal gas constant (8.314 J mol^−1^ K^−1^); *T* (K) represents the absolute temperature; and *A* (L g^−1^) and *B* (J mol^−1^) are the Temkin constants.

#### 2.3.3 Adsorption kinetics experiments

Samples were collected at different adsorption times (1, 5, 10, 15, 20, 30, 60, 120, 240, 480, 720, and 1440 min). The initial pollutant concentrations were set at the maximum values listed in Table 2, while all other experimental conditions were maintained constant (pH = 7, 180 rpm, 25 °C, 1.60 g per L P-HAP). The adsorption kinetics experiments were conducted to investigate the adsorption process of PTEs by P-HAP in aqueous solution.

The adsorption kinetics data were fitted using the pseudo-first-order (7) and pseudo-second-order (8) kinetic models. In addition, the intra-particle diffusion model (9) was applied to further analyze the adsorption mechanism of metal pollutants by P-HAP. The corresponding equations are as follows:

7 *Q*_*t*_ = *Q*_e_(1 − e^−*k*_1_*t*^)

8
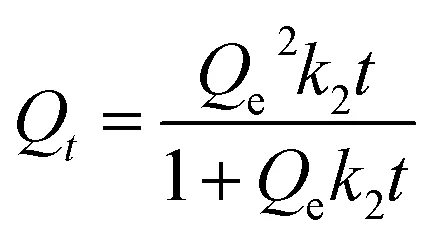

9 *Q*_*t*_ = *k*_id_*t*^0.5^ + *C*

where *Q*_*t*_ (mg g^−1^) and *Q*_e_ (mg g^−1^) represent the adsorption capacities of P-HAP for the pollutant at time *t* and at equilibrium, respectively; *k*_1_ (min^−1^) and *k*_2_ (min^−1^) are the rate constants for the pseudo-first-order and pseudo-second-order kinetic models, respectively; *k*_id_ (mg (g^−1^ min^−1/2^)) is the intraparticle diffusion rate constant; and *C* represents the intercept related to the boundary layer effect and reflects the thickness of the external diffusion layer.

### 2.4 Data processing

The experimental data were statistically analyzed using SPSS 24.0. The phase composition and synthesis rate were calculated and analyzed using MDI JADE 6.0 and XPowder_01.32. The specific surface area and pore size distribution of the samples were determined using the corresponding calculation models. The specific surface area was calculated using the Brunauer–Emmett–Teller (BET) model, while the pore size distribution was obtained using the Barrett–Joyner–Halenda (BJH) model based on the desorption isotherm. FTIR spectra were analyzed using EZ OMNIC 7.3, and all figures were generated using Origin 2024.

## 3 Results and discussion

### 3.1 Preparation and structural characterization of P-HAP

**Fig. 2:**
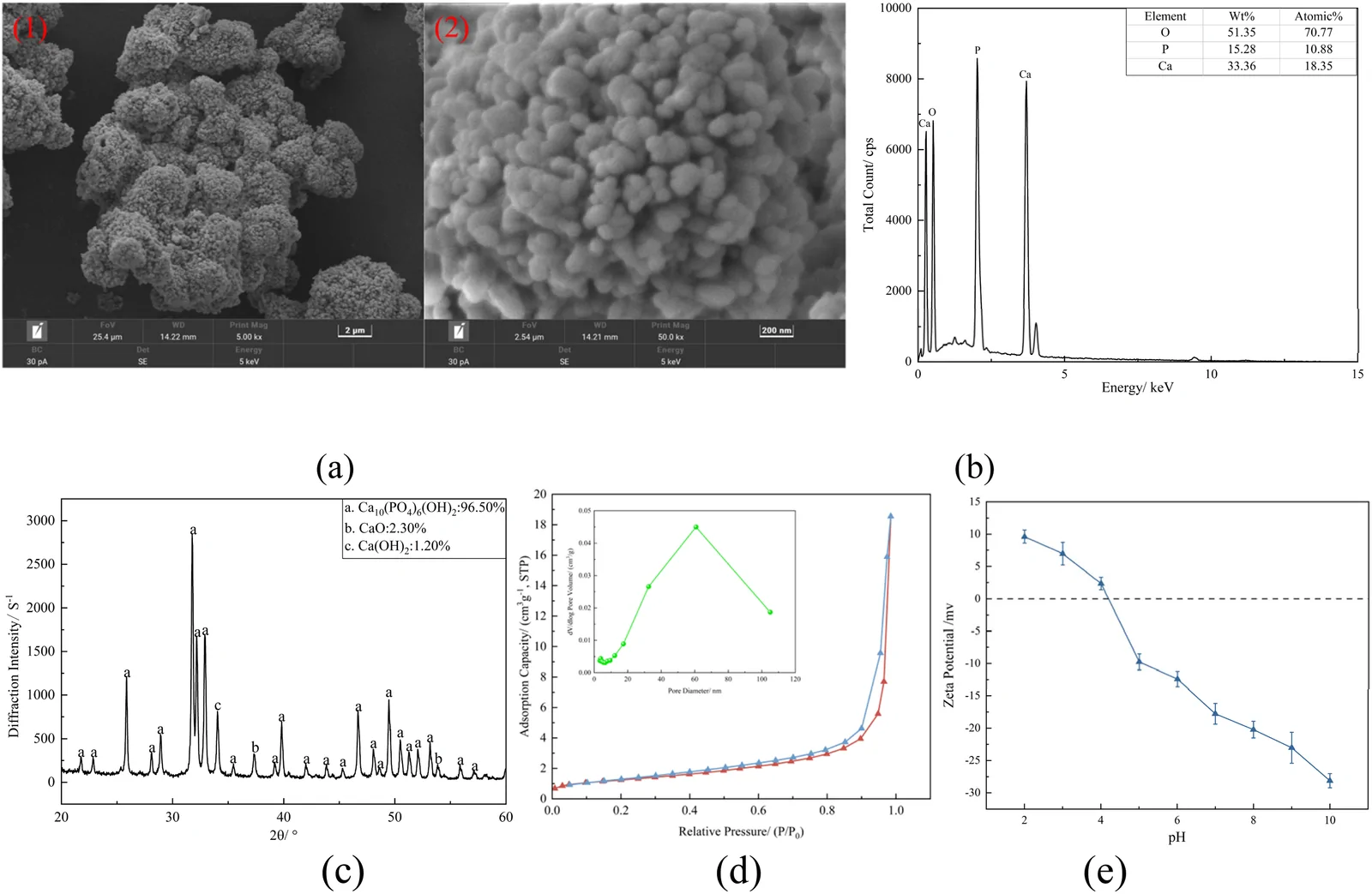
Characterization of P-HAP: (a) SEM ((1) 2 µm (2) 200 nm) (b) EDS (c) XRD phase analysis diagram (d) BJH pore size distribution diagram and nitrogen adsorption–desorption isothermal diagram (e) zeta potential variation of P-HAP at pH 2–10.

The P-HAP contents determined by XRD analysis were 94.8%, 95.5%, and 95.9%, respectively, with an average phase purity of 95.4%. The microscopic morphology of P-HAP was observed using scanning electron microscope, as shown in Fig. 2(a). The individual particles exhibit diameters of approximately 75–100 nm and form rough, porous spherical aggregates.^19^ Elemental analysis by EDS (Fig. 2(b)) revealed that the sample consists primarily of calcium (Ca), phosphorus (P), and oxygen (O). The measured calcium–phosphorus atomic ratio (Ca/P) was approximately 1.689, which is close to the theoretical stoichiometric ratio of 1.67 for hydroxyapatite. Fig. 2(c) presents the XRD pattern of P-HAP. The diffraction peaks of the synthesized P-HAP were highly consistent with those of the standard HAP diffraction pattern (JCPDS no. 09-0432),^20^ confirming that the main crystalline phase of the product was HAP crystal.

Fig. 2(d) shows the BJH pore size distribution and nitrogen adsorption–desorption isotherm of P-HAP. The results indicate that the specific surface area, pore volume, and average pore size of P-HAP are 4.395 m^2^ g^−1^, 0.0287 cm^3^ g^−1^, and 26.09 nm, respectively. The adsorption–desorption isotherm of P-HAP gradually increases with increasing relative pressure and eventually reaches a plateau, exhibiting the characteristics of a type IV isotherm, which indicates that P-HAP is a mesoporous material.^21^ The hysteresis loop is classified as type H3, which is commonly associated with slit-shaped pores and aggregates of particles, consistent with the morphological characteristics of HAP. Furthermore, the relatively wide hysteresis loop suggests the presence of abundant mesoporous structures in the prepared P-HAP, which agrees well with the SEM observations. As shown in Fig. 2(e), the zeta potential of P-HAP dispersed in solution after ultrasonication for 2 h was measured using a potentiometer. The point of zero charge (pH_pzc_) of P-HAP was determined to be 4.198. When the solution pH is lower than the pH_pzc_, the surface of P-HAP becomes positively charged, resulting in electrostatic repulsion toward positively charged metal ions and promoting the adsorption of anions. Conversely, when the pH exceeds the pH_pzc_, the surface becomes negatively charged, which facilitates the adsorption of cationic species. With increasing pH, the dispersion stability of the solution initially decreases and then increases, reaching a higher stability level.

### 3.2 Effects of P-HAP dosage

**Fig. 3:**
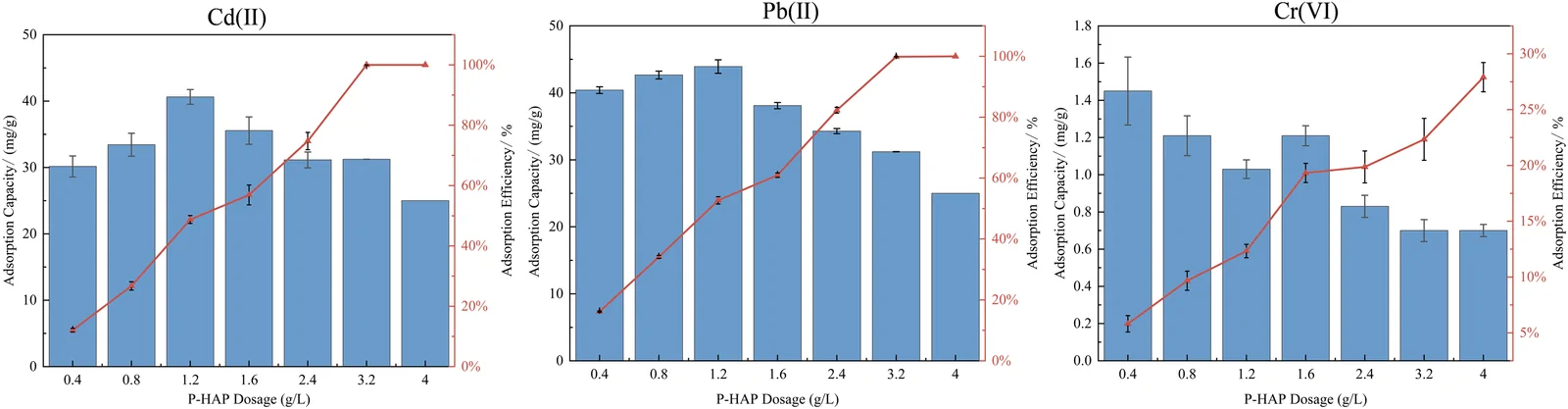
Effects of P-HAP dosage on the adsorption of three PTEs.

The dosage of adsorbent is a critical factor affecting the adsorption performance and removal efficiency of pollutants in aqueous solutions. Fig. 3 illustrates the relationship between P-HAP dosage and the adsorption capacity and removal efficiency of different pollutants. At a P-HAP dosage of 1.20 g L^−1^, the maximum adsorption capacities of P-HAP for Cd(ii) and Pb(ii) reached 40.63 mg g^−1^ and 43.92 mg g^−1^, respectively. For Cr(vi), the maximum adsorption capacity of 1.45 mg g^−1^ was obtained at a P-HAP dosage of 0.40 g L^−1^.

With increasing P-HAP dosage from 0.40 to 4.00 g L^−1^, the removal efficiencies of Cd(ii) and Pb(ii) increased from 12.07% and 16.16% to 100%, respectively. Meanwhile, the removal efficiency of Cr(vi) increased from 5.81% to 27.91%. Cd(ii) and Pb(ii) could be completely removed at a P-HAP dosage of 3.20 g L^−1^, whereas complete removal of Cr(vi) was not achieved. These results indicate that increasing the P-HAP dosage had a limited effect on the adsorption capacity of Cr(vi), and its overall adsorption efficiency remained relatively low.

With increasing P-HAP dosage, the adsorption capacity of P-HAP for all PTEs generally decreased, whereas the removal efficiency increased. This phenomenon can be attributed to the fact that, when the initial concentration of PTEs remains constant, increasing the amount of P-HAP provides more available adsorption sites, thereby facilitating the transfer of contaminants from the aqueous phase to the adsorbent surface and reducing the residual PTE concentration in the solution. Consequently, the removal efficiency increases. However, the same amount of PTEs is distributed among a larger mass of adsorbent, resulting in fewer PTEs adsorbed per unit mass of P-HAP and, consequently, a decrease in adsorption capacity.^22^

### 3.3 Effects of solution pH

**Fig. 4:**
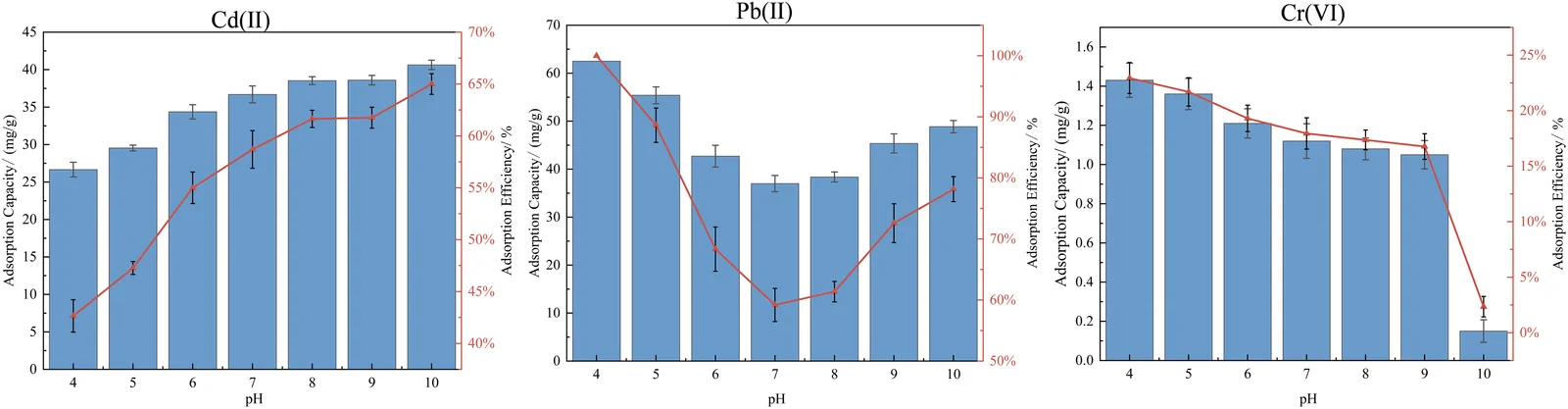
Effect of solution pH on adsorption of three PTEs.

Solution pH is an important factor affecting the surface charge properties of P-HAP and, consequently, the adsorption behavior of PTEs in aqueous solutions. The relationship between solution pH and the adsorption capacity and removal efficiency of pollutants is shown in Fig. 4. The adsorption capacity and removal efficiency of P-HAP for Cd(ii) increase with increasing pH and reach their maximum values at pH 10, with values of 40.63 mg g^−1^ and 65.00%, respectively. In contrast, the adsorption capacity and removal efficiency of P-HAP for Pb(ii) initially decreased and then increased with increasing pH, reaching their maximum values at pH 4, with an adsorption capacity of 62.50 mg g^−1^ and a removal efficiency of 100%. For Cr(vi), both the adsorption capacity and removal efficiency decreased as the solution pH increased, with the highest values obtained at pH = 4 (1.43 mg g^−1^ and 22.94%, respectively).

The zeta potential results indicate that when the solution pH exceeds the pH_pzc_ (4.198), the surface of P-HAP becomes negatively charged, which is favorable for the adsorption of positively charged Pb(ii) and Cd(ii) ions. In contrast, when the pH is lower than the pH_pzc_ (4.198), the positively charged P-HAP surface repels cationic PTEs but promotes the adsorption of negatively charged Cr_2_O_7_^2−^ anions through electrostatic attraction. Under alkaline conditions, Cd(ii) can react with OH^−^ in solution to form Cd(OH)_2_ precipitates.^23^ Therefore, alkaline conditions are more favorable for the adsorption and removal of Cd(ii), whereas acidic conditions are more favorable for that of Cr(vi). Under strongly acidic conditions, P-HAP undergoes dissolution, releasing PO_4_^3−^ species that react with Pb(ii) to form insoluble lead phosphate precipitates. Therefore, the adsorption of Pb(ii) under acidic conditions is primarily governed by the dissolution-precipitation mechanism. When the solution pH ranges from 5 to 7, the dissolution of P-HAP is suppressed, weakening the precipitation process. Meanwhile, competitive adsorption between H^+^ and metal ions for surface adsorption sites occurs, leading to a decrease in adsorption capacity.^24^ Therefore, acidic conditions are more favorable for the adsorption and removal of Pb(ii).

### 3.4 Adsorption isotherm analysis

The fitting results of the three adsorption isotherm models for Cd(ii), Pb(ii), and Cr(vi) adsorption onto P-HAP are presented in Table 3. The correlation coefficients (*R*^2^) obtained from the Langmuir model were higher than those of the Freundlich and Temkin models, while the residual sum of squares (SSE) and root mean square error (RMSE) of the Langmuir model were lower than those of the Freundlich and Temkin models, indicating that the Langmuir model provided a better description of the adsorption process. This suggests that the adsorption of PTEs mainly occurred on relatively homogeneous active sites of the P-HAP surface. For all three PTEs, the adsorption process was primarily characterized as monolayer adsorption.^25^

**Table 3:** Parameters of the adsorption isotherm model of P-HAP for three PTEs

Adsorption isotherm model	Fitting parameters	Cd(ii)	Pb(ii)	Cr(vi)
Langmuir	*Q*_m_ (mg g^−1^)	53.39	73.86	1.72
	*K*_L_ (L mg^−1^)	5.37	16.86	11.48
	*R* ^2^	0.973	0.984	0.967
	SSE	1.649	8.051	0.060
	RMSE	0.406	0.897	0.077
Freundlich	*K*_F_ (mg g^−1^ (L mg^−1^)^1/*n*^)	42.94	90.28	1.29
	1/*n*	0.078	0.323	0.182
	*R* ^2^	0.513	0.873	0.864
	SSE	30.143	65.525	0.251
	RMSE	1.736	2.560	0.158
Temkin	*A* (L g^−1^)	78 061.21	171.87	544.99
	*B* (J mol^−1^)	3.81	15.70	0.22
	*R* ^2^	0.590	0.890	0.932
	SSE	944.467	319.425	0.125
	RMSE	9.718	5.652	0.112

The Langmuir adsorption equilibrium constant (*K*_L_) reflects the affinity between the adsorbent and adsorbate; a higher *K*_L_ value indicates stronger interaction between the adsorbent and the adsorbate. The affinity order of P-HAP toward the three PTEs was *K*_LPb(II)_ > *K*_LCr(VI)_ > *K*_LCd(II)_. Furthermore, at the same initial concentration, the separation factor (*R*_L_) followed the order Pb(ii) < Cr(vi) < Cd(ii), indicating that P-HAP exhibited the strongest adsorption affinity toward Pb(ii). This result is consistent with previous studies reported by Mobasherpour *et al.*^14^ and Shen *et al.*^26^ According to the Langmuir model, the theoretical maximum adsorption capacities (*Q*_m_) of P-HAP for Cd(ii), Pb(ii), and Cr(vi) were estimated to be 53.39 mg g^−1^, 73.86 mg g^−1^, and 1.72 mg g^−1^, respectively.

**Fig. 5:**
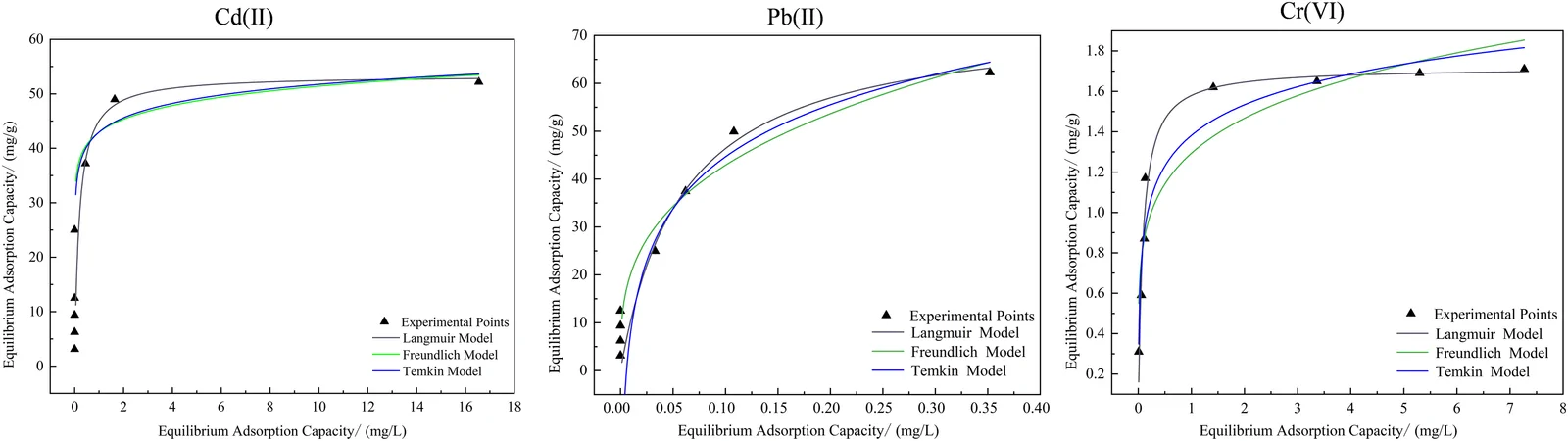
Adsorption isotherms of P-HAP for three PTEs.

As shown in Fig. 5, the Langmuir isotherms of the three PTEs increased rapidly at low equilibrium concentrations and gradually approached a plateau with further increases in concentration, exhibiting the typical characteristics of Langmuir monolayer adsorption. At low concentrations, sufficient adsorption sites are available, and the adsorption capacity increases rapidly with increasing concentration. As the concentration continues to increase, the active sites are gradually occupied, the adsorption rate decreases, and the increase in adsorption capacity slows down. At high concentrations, the adsorption sites gradually become saturated, and the adsorption capacity approaches the maximum adsorption capacity (*Q*_m_).

### 3.5 Adsorption kinetics analysis

To better understand the adsorption process of PTEs in aqueous solutions, batch experiments were conducted to evaluate the effect of adsorption time on the adsorption of PTEs by P-HAP. The experimental data were then nonlinearly fitted using the pseudo-first-order, pseudo-second-order, and intraparticle diffusion kinetic models.

**Fig. 6:**
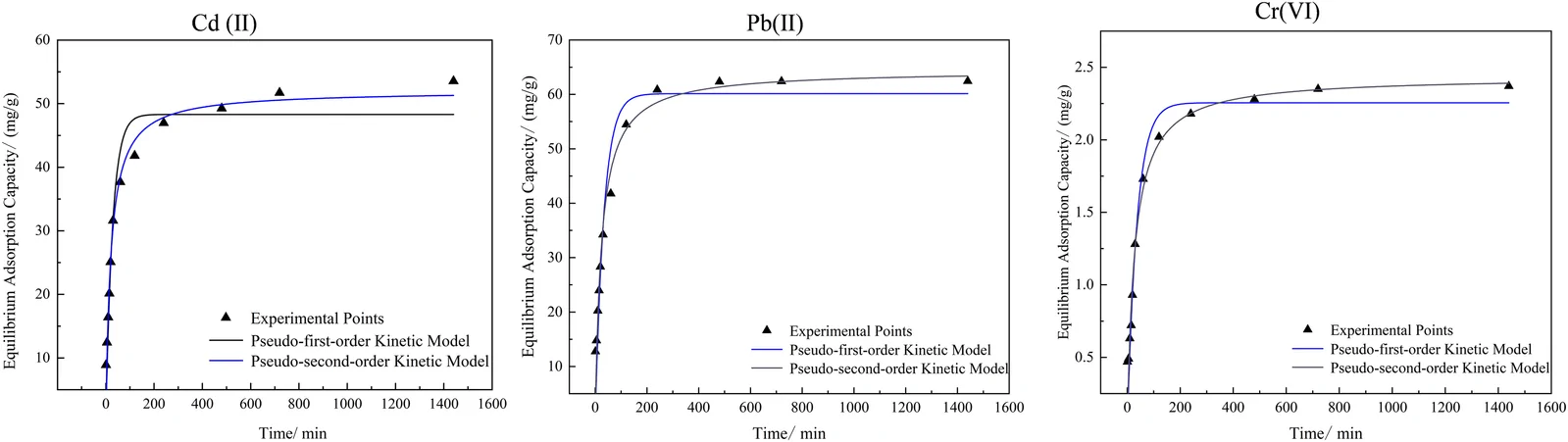
Model of adsorption kinetics of P-HAP for three PTEs.

The *R*^2^ of the pseudo-second-order kinetic model were higher than those of the pseudo-first-order model, while the SSE and RMSE of the pseudo-second-order model were lower, as shown in Table 4. The pseudo-second-order model provided a better description of the experimental data.^27,28^ As shown in Fig. 6, the adsorption capacity of P-HAP for Cd(ii) increased rapidly during the first 1–60 min, and the rate of increase slowed after 60 min. The adsorption of Cd(ii) reached equilibrium at 720 min, with a removal efficiency of 85.67%. The adsorption capacities of P-HAP for Pb(ii) and Cr(vi) increased rapidly during the first 1 to 120 min, after which the rate of increase gradually slowed. The adsorption of Pb(ii) reached equilibrium at 480 min, whereas that of Cr(vi) reached equilibrium at 720 min. Ultimately, the removal efficiencies of Pb(ii) and Cr(vi) reached 99.90% and 28.00%, respectively.

**Table 4:** Parameters of the adsorption kinetics model of PTEs in water by P-HAP

Adsorption kinetics model	Fitting parameters	Cd(ii)	Pb(ii)	Cr(vi)
Pseudo-first-order dynamics model	*Q*_e_ (mg g^−1^)	48.309	60.136	2.256
	*k*_1_ (min^−1^)	0.036	0.030	0.027
	*R* ^2^	0.932	0.926	0.954
	SSE	176.417	294.218	0.28
	RMSN	3.834	4.952	0.152
Pseudo-second-order dynamics model	*Q*_e_ (mg g^−1^)	52.049	64.444	2.437
	*k*_2_ (min^−1^)	9.062 × 10^−4^	6.404 × 10^−4^	0.015
	*R* ^2^	0.974	0.963	0.968
	SSE	66.354	148.151	0.20
	RMSN	2.351	3.514	0.126
In-particle diffusion model	*k*_id,1_ mg (g^−1^ min^−1/2^)	4.630	4.679	0.105
	*C*_1_	3.249	6.457	0.343
	*R* ^2^	0.966	0.973	0.911
	*k*_id,2_ mg (g^−1^ min^−1/2^)	0.816	2.395	0.023
	*C*_2_	32.488	25.071	1.161
	*R* ^2^	0.888	0.840	0.853
	*k*_id,3_ mg (g^−1^ min^−1/2^)	0.249	0.060	0.005
	*C*_3_	44.302	60.482	1.552
	*R* ^2^	0.819	0.318	0.449

**Fig. 7:**
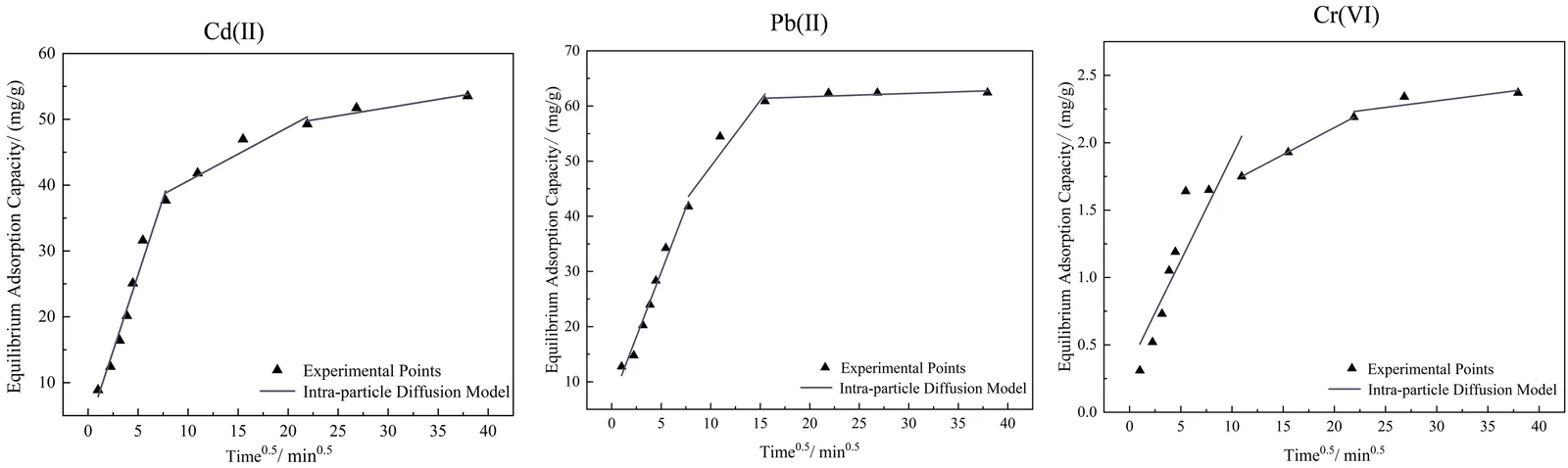
Model of intraparticle diffusion of three PTEs by P-HAP.

Fig. 7 describes the intraparticle diffusion behavior of metal ions during adsorption by P-HAP. The slope constant (*k*) for each metal ion gradually decreased over the three stages (*k*_1_ > *k*_2_ > *k*_3_), while the intercept constant (*C*) gradually increased (*C*_1_ < *C*_2_ < *C*_3_). At the initial stage of adsorption, abundant adsorption sites were available on the surface of P-HAP, allowing PTEs to be rapidly adsorbed and resulting in the highest adsorption rate. As adsorption proceeded, the number of available adsorption sites gradually decreased, leading to a reduction in the adsorption rate. When the adsorption sites on the P-HAP surface were nearly occupied by PTEs, the adsorption process reached equilibrium. The curves in Fig. 7 did not pass through the origin (*i.e.*, the intercept constant *C* ≠ 0), indicating that the adsorption of metal ions by P-HAP was governed by both intraparticle diffusion and external surface adsorption. Therefore, the adsorption of metal ions by P-HAP can be regarded as a multi-stage adsorbent–adsorbate interaction process, with the diffusion stage serving as the primary rate-controlling step.^29^

### 3.6 Analysis of adsorption mechanisms of Cd(ii), Pb(ii), and Cr(vi) by P-HAP

**Fig. 8:**
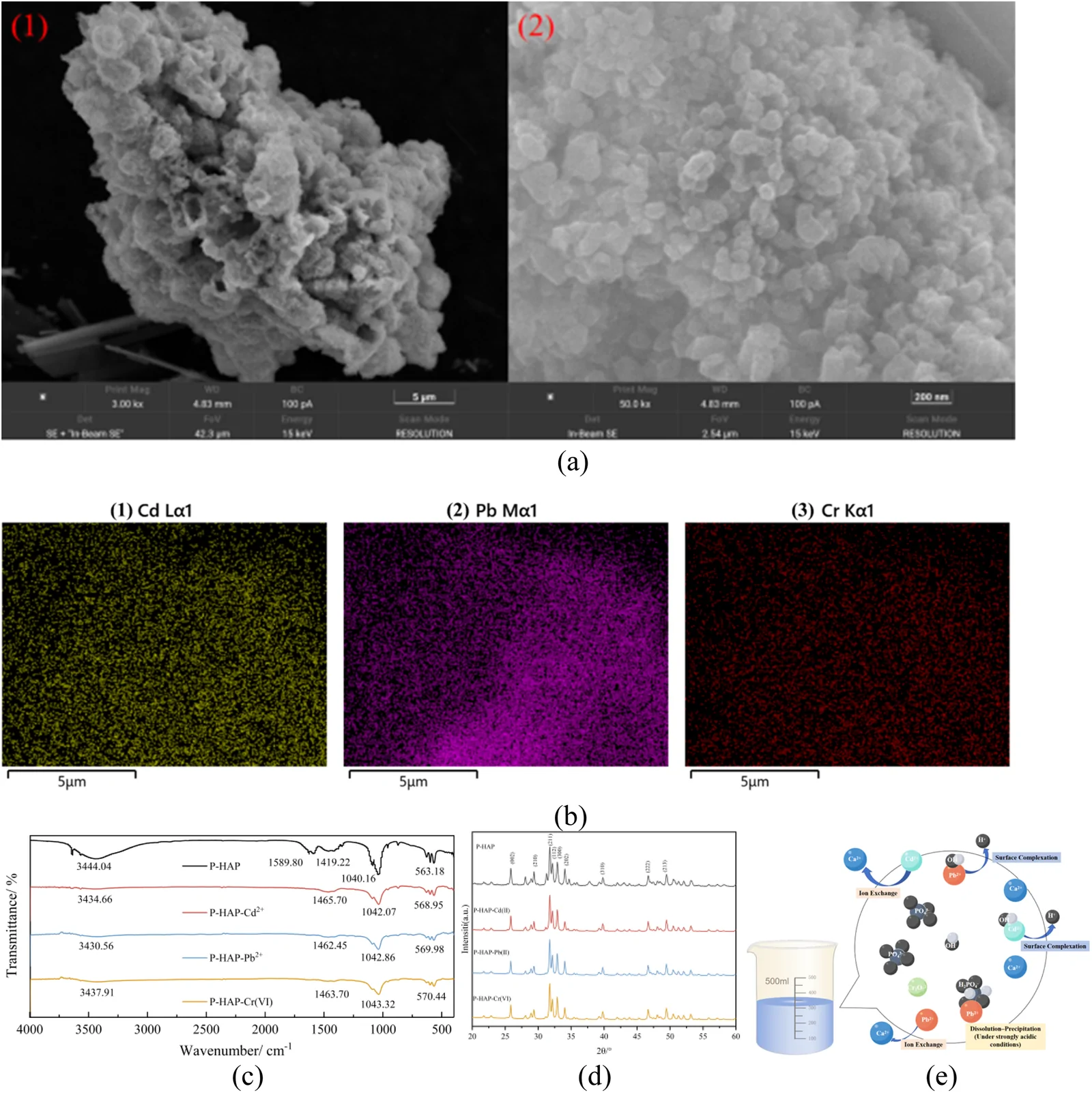
Characterization of the post-adsorption sample: (a) SEM images ((1) 5 µm, (2) 200 nm), (b) EDS spectrum, (c) FTIR spectra comparison of P-HAP before and after adsorption, (d) XRD patterns of P-HAP before and after adsorption, (e) schematic diagram of the adsorption mechanism.

In Fig. 2(a), pristine P-HAP before adsorption exhibits sphere-like agglomerates with rough surfaces and abundant pores. After adsorption, the surface morphology of P-HAP shown in Fig. 8(a) reveals flake-like or block-like deposits, with smoother particle surfaces, particle aggregation, and pore-filling phenomena observed.^30^ The EDS results presented in Fig. 8(b) confirm the presence of Cd, Pb, and Cr elements on the surface of P-HAP after adsorption, indicating that all three PTEs were successfully adsorbed onto the P-HAP surface. Fig. 8(c) shows the FTIR spectra of P-HAP before and after the adsorption of Cd(ii), Pb(ii), and Cr(vi). The –OH stretching vibration peak of P-HAP at 3444.04 cm^−1^ shifted and decreased in intensity after PTE adsorption, indicating that complexation reactions occurred between the PTEs and surface –OH groups.^31^ Following the adsorption of Cd(ii), Pb(ii), and Cr(vi), the characteristic absorption peaks of PO_4_^3−^ at 1040.16 cm^−1^ and 563.18 cm^−1^ exhibited shifts accompanied by reduced intensities, suggesting that surface complexation and dissolution–precipitation processes occurred between the PTEs and PO_4_^3−^.^32,33^ The asymmetric stretching vibration peak of CO_3_^2−^ at 1419.22 cm^−1^ also shifted to varying degrees after PTE adsorption, indicating the involvement of carbonate groups in surface complexation reactions with the PTEs.^34^ Furthermore, the H–O–H bending vibration peak of adsorbed water at 1589.80 cm^−1^ disappeared after adsorption, suggesting that Cd(ii), Pb(ii), and Cr(vi) occupied the active sites on the P-HAP surface, thereby disrupting the hydrogen bonding interactions between adsorbed water molecules and surface functional groups.

The XRD results (Fig. 8(d)) further reveal the changes in the crystal structure of P-HAP during the adsorption process. After the adsorption of Cd(ii) and Pb(ii), the characteristic diffraction peaks of P-HAP exhibited slight shifts accompanied by varying degrees of intensity reduction. Specifically, the main diffraction peak (211) shifted from 2*θ* = 31.80° to 31.78° and 31.76° after Cd(ii) and Pb(ii) adsorption, respectively. These shifts may be attributed to the ion exchange between Cd(ii)/Pb(ii) and Ca^2+^ within the P-HAP lattice, resulting in localized lattice distortion. Among them, Pb(ii) has a larger ionic radius than Ca^2+^; therefore, its substitution for Ca^2+^ induces more pronounced lattice distortion. In contrast, the ionic radius of Cd(ii) is closer to that of Ca^2+^, leading to relatively weaker structural perturbation. Cr(vi) mainly exists in anionic forms, such as Cr_2_O_7_^2−^, which limits its ability to undergo ion exchange with Ca^2+^ in the P-HAP lattice. Therefore, the removal of Cr(vi) mainly occurs through surface complexation mechanisms, resulting in the least noticeable changes in the XRD diffraction patterns.

Combined with the zeta potential results shown in Fig. 2(e), the adsorption behavior of P-HAP is strongly influenced by the solution pH. When the solution pH is higher than pH_pzc_ (4.198), the P-HAP surface becomes negatively charged, favoring the adsorption of positively charged Cd(ii) and Pb(ii). Conversely, when the solution pH is lower than pH_pzc_ (4.198), the P-HAP surface is positively charged, which repels cationic PTEs while promoting the adsorption of negatively charged Cr(vi) species. Conversely, the adsorption mechanisms of P-HAP for different PTEs vary with solution pH. Fig. 8(e) schematically illustrates the proposed adsorption mechanisms. Under acidic conditions, P-HAP partially dissolves, releasing Ca^2+^ and PO_4_^3−^ into the solution. At this stage, the adsorption of Pb(ii) is dominated by the dissolution–precipitation mechanism, in which Pb(ii) reacts with PO_4_^3−^ to form insoluble lead phosphate precipitates (*e.g.*, Pb_5_(PO_4_)_3_OH),^26^ while ion exchange also contributes synergistically (reactions (1) and (2)). As the solution pH increases, the dissolution of P-HAP is suppressed, and surface –OH and PO_4_^3−^ become the primary active sites. Under these conditions, Pb(ii) is predominantly immobilized through surface complexation (reaction (3)),^31^ with ion exchange continuing to contribute to the overall adsorption process. Under acidic conditions, the adsorption of Cd(ii) by P-HAP is dominated by ion exchange, with a portion of Cd(ii) combining with PO_4_^3−^ to form cadmium phosphate compounds (reactions (1) and (2)).^26^ Under neutral and alkaline conditions, the adsorption of Cd(ii) is jointly controlled by surface complexation and ion exchange (reactions (1) and (3)).^23,35^ In contrast, Cr(vi) predominantly exists as anionic species, such as Cr_2_O_7_^2−^ and HCrO_4_^−^, and therefore exhibits an adsorption behavior markedly different from those of Cd(ii) and Pb(ii). The ionic species of Cr(vi) in solution depend on the pH value. In the pH range of 4–6.5, HCrO_4_^−^ is the predominant species, while at pH > 6.5, Cr_2_O_7_^2−^ becomes dominant. Because of its anionic nature, Cr(vi) is unable to participate in ion exchange with Ca^2+^, and its removal is mainly attributed to surface complexation with the functional groups present on the P-HAP surface.^36^ Overall, the adsorption and removal of PTEs by P-HAP are primarily governed by three mechanisms: ion exchange, dissolution–precipitation, and surface complexation, which can be described by the following chemical reactions:^26,37^

(1) Ion exchange: Ca^2+^ in P-HAP is replaced by PTEs:^38^Ca_5_(PO_4_)_3_OH + *x*M^*n*+^ → Ca_5−*x*_M_*x*/*n*_(PO_4_)_3_OH + *x*Ca^2+^

(2) Dissolution–precipitation: P-HAP dissolves under acidic conditions to form new insoluble substances with PTEs:^39^Ca_5_(PO_4_)_3_OH + 7H^+^ → 3H_2_PO_4_^−^ + 5Ca^2+^ + H_2_O3H_2_PO_4_^−^ + 5M^2+^ + H_2_O → M_5_(PO_4_)_3_OH↓ + 7H^+^

(3) Surface complexation: metal ions in the solution are adsorbed onto the HAP surface through coordination.^31^HAP–OH + M^*n*+^ ↔ HAP–O–M^(*n*−1)+^ + H^+^2HAP–OH + M^*n*+^ ↔ (HAP–O)_2_–M^(*n*−2)+^ + 2H^+^

### 3.7 Comparison of P-HAP with other adsorbents

Using waste putty powder from construction waste as the calcium source, this study prepared hydroxyapatite (P-HAP) *via* chemical precipitation. The raw material is widely available and low in cost, while simultaneously achieving resource utilization of construction waste. Compared with other typical adsorbents listed in Table 5, P-HAP exhibits excellent adsorption performance for the removal of Pb(ii) and Cd(ii), showing certain application potential; however, its adsorption efficiency for Cr(vi) is still unsatisfactory and requires further modification and optimization.

**Table 5:** Comparison of adsorption performance of different typical HAP-based adsorbents for Pb(ii), Cd(ii), and Cr(vi)

Adsorbents	Adsorption capability (mg g^−1^)	pH	Dose (mg L^−1^)	Contact time (min)	Regeneration
Shell-derived hydroxyapatite^40^	Pb(ii): 20.0	7	4	60	The removal rates for lead, chromium, and cadmium reached 109.88%, 51.68%, and 76.02%, respectively
	Cd(ii): 2.5				
	Cr(vi): 9.5				
Eggshell-derived hydroxyapatite^41^	Cr(vi): 0.26	5.7	4	20	Not conducted
Walnut shells^42^	Pb(ii): 6.82	5	10	120	Not conducted
	Cd(ii): 4.36				
HAp/Nap nanocomposite^43^	Pb(ii): 55.55	6	1	120	In acidic solution, after 10 adsorption/desorption cycles, the desorption rates of adsorbed Pb(ii) and Cd(ii) reached approximately 70% and 64%, respectively
	Cd(ii): 40.16				
HAP^44^	Pb(ii): 15.08	5	0.4	Pb: 130	When using 0.1 M HCl for desorption, the initial desorption efficiencies were 79.7% for Cd^2+^ and 68.32% for Pb^2+^, and the material remained stable during four cycles of repeated use
	Cd(ii): 20.24			Cd: 120	
P-HAP (this study)	Pb(ii): 62.50	Pb(ii): 4	1.6	Pb: 480	Not conducted
	Cd(ii): 40.63	Cd(ii): 10		Cr, Cd: 720	
	Cr(vi): 1.43	Cr(vi): 4			

### 3.8 Environmental friendliness and economic feasibility analysis

Compared with the conventional synthesis of hydroxyapatite using analytical-grade calcium salts, this study utilized waste putty powder, a construction waste by-product, as the calcium source for the preparation of P-HAP. As an abundant solid waste generated during building demolition and renovation activities, waste putty powder represents a sustainable and cost-effective alternative calcium source. Its utilization not only promotes the valorization of construction waste but also reduces the consumption of natural calcium resources, thereby supporting resource recovery and aligning with the principles of the circular economy.

P-HAP was synthesized using a chemical precipitation method without the use of toxic organic solvents or complex post-treatment processes. However, the preparation still involves high-temperature calcination, which inevitably results in significant energy consumption. Therefore, the environmental and economic advantages of this approach are mainly attributed to the utilization of waste-derived raw materials. A comprehensive evaluation based on life cycle assessment (LCA) and techno-economic assessment (TEA) is needed to quantitatively assess the overall environmental impacts and economic feasibility of the proposed method, particularly for large-scale production and practical applications.

## 4 Conclusions and perspectives

### 4.1 Conclusions

(1) P-HAP was successfully synthesized *via* a chemical precipitation method using waste putty powder derived from construction debris as the calcium source, achieving an average phase purity of 95.4%. The results indicate that waste putty powder can serve as an effective alternative calcium source for hydroxyapatite synthesis, providing a sustainable approach for the resource utilization of construction waste.

(2) P-HAP exhibited distinctly different adsorption behaviors toward the investigated potentially toxic metal ions, with the overall adsorption performance following the order: Pb(ii) > Cd(ii) > Cr(vi). Among these, the maximum adsorption capacity for Pb(ii) reached 62.50 mg g^−1^, achieving complete removal under the experimental conditions, whereas the maximum adsorption capacity for Cd(ii) was 40.63 mg g^−1^. In contrast, the maximum adsorption capacity for Cr(vi) was only 1.43 mg g^−1^, indicating that P-HAP exhibits a substantially higher adsorption affinity for cationic metal ions than for anionic Cr(vi) species.

(3) Adsorption isotherm and kinetic analyses indicated that the adsorption behaviors of Pb(ii), Cd(ii), and Cr(vi) on P-HAP are better described by the Langmuir isotherm model and the pseudo-second-order kinetic model. Combined with the XRD, SEM, EDS, FTIR, and zeta potential analyses, the results demonstrate that the removal of Pb(ii) and Cd(ii) is primarily governed by dissolution–precipitation, ion exchange, and surface complexation. In contrast, the adsorption of Cr(vi) is dominated by surface complexation, with electrostatic interactions playing a synergistic role in the overall adsorption process.

### 4.2 Perspectives

(1) Future research should focus on the desorption behavior, leaching characteristics, regeneration, and recyclability of P-HAP. Systematic investigations should be conducted to evaluate the risk of heavy metal re-release under different environmental conditions, as well as the adsorption and structural stability of P-HAP after repeated adsorption–desorption cycles. Such studies are essential to verify its long-term applicability, reusability, and environmental safety.

(2) Since P-HAP may partially dissolve under certain environmental conditions, leading to the release of phosphate ions, this process should be considered a potential source of secondary pollution. Future studies should quantitatively investigate phosphate release under different hydrochemical conditions and combine these results with ecological risk assessment methods to comprehensively evaluate its potential environmental impact.

## Author contributions

All the authors contributed to the ideas and methodology. This research was completed jointly by all the authors. The specific contributions are as follows: Yonghong Zheng was responsible for the conceptualization, funding acquisition, investigation, data collection, project administration, resources, writing – original draft and editing of the paper; Ziyang Zhang was responsible for the investigation, statistical analysis, writing – editing the manuscript and data curation; Zhiguo Zhang (corresponding author) was responsible for the conceptualization, writing – editing and reviewing the manuscript; Weihua Peng responsible for the funding acquisition, investigation, project administration; and Yi Liu, Xueting Zhen, and Mingxia Huang were responsible for data organization and investigation. All the authors read and finalized the manuscript.

## Conflicts of interest

The authors declare that they have no competing interests.

## Data Availability

Data supporting the findings of this study are available from the corresponding author upon reasonable request.
